# Assessing Surgical Approaches and Postoperative Complications for Thoracic Schwannomas: A Multicenter Retrospective Observational Analysis of 106 Cases

**DOI:** 10.3390/cancers17071177

**Published:** 2025-03-31

**Authors:** Giuseppe Corazzelli, Antonio Bocchetti, Marco Filippelli, Maria Marvulli, Sergio Corvino, Valentina Cioffi, Vincenzo Meglio, Settimio Leonetti, Ciro Mastantuoni, Maria Rosaria Scala, Alberto de Bellis, Alessandra Alfieri, Roberto Tafuto, Francesco Ricciardi, Salvatore Di Colandrea, Alessandro D’Elia, Luigi Sigona, Mauro Mormile, Pasqualino De Marinis, Sergio Paolini, Vincenzo Esposito, Alfonso Fiorelli, Gualtiero Innocenzi, Raffaele de Falco

**Affiliations:** 1 Neurosurgery Department, Santa Maria delle Grazie Hospital, ASL Napoli 2 Nord, 80078 Naples, Italy; antonio.bocchetti74@gmail.com (A.B.); valecioffi81@gmail.com (V.C.); mastantuoniciro@gmail.com (C.M.); drscala.mariarosaria@gmail.com (M.R.S.); doct13@libero.it (L.S.); raffaele.defalco@aslnapoli2nord.it (R.d.F.); 2 Division of Neurosurgery, Department of Neurosciences, Reproductive and Odontostomatological Sciences, “Federico II” University, 80131 Naples, Italy; sercorvino@gmail.com; 3 Department of Clinical Medicine and Surgery, University of Naples Federico II, Via S. Pansini 5, 80131 Naples, Italy; marcofilippelli94@gmail.com (M.F.); mauro.mormile@unina.it (M.M.); 4 Thoracic Surgery Unit, Department of Translational Medicine, Università degli Studi della Campania “Luigi Vanvitelli”, 80122 Naples, Italy; mariamarvulli.m@libero.it (M.M.); alfonso.fiorelli@unicampania.it (A.F.); 5 Department of Neurosurgery, AORN Sant’Anna e San Sebastiano, 81100 Caserta, Italy; vinmeglio790@gmail.com (V.M.); albertodebellis@hotmail.com (A.d.B.); alessandralfieri@yahoo.it (A.A.); rob.tafuto@gmail.com (R.T.); pasqualino.demarinis@gmail.com (P.D.M.); 6 Department of Neurosurgery, IRCCS Neuromed, 86077 Pozzilli, Italy; settimioleonetti@gmail.com (S.L.); fricciardi1@yahoo.it (F.R.); deliaale@gmail.com (A.D.); sergio.paolini@uniroma1.it (S.P.); vincenzo.esposito@uniroma1.it (V.E.); innocenzigualtiero@tiscali.it (G.I.); 7 Department of Anaesthesiology and Intensive Care Medicine, Santa Maria delle Grazie Hospital, ASL Napoli 2 Nord, 80078 Naples, Italy; salvatore.dicolandrea@aslnapoli2nord.it

**Keywords:** thoracic schwannoma, spinal nerve sheath tumors, Eden classification, minimally invasive spine surgery, video-assisted thoracoscopic surgery (VATS), gross total resection (GTR), surgical complications

## Abstract

**Background:** Thoracic schwannomas are benign nerve sheath tumors that can cause neurological and respiratory symptoms depending on their location and extension. The optimal surgical approach remains debated, particularly regarding resection extent, complication rates, and postoperative morbidity. **Methods:** This retrospective multicenter study analyzed 106 patients treated between 2011 and 2024, classifying tumors according to the Eden system and comparing surgical strategies. Surgical variables, including operative time, blood loss, resection extent, recurrence rates, and complications classified by Clavien–Dindo, were analyzed. **Results:** Eden I and II schwannomas were treated with laminectomy (LCT) or hemilaminectomy (HLCT) and transpedicular approaches (TPD), achieving high gross total resection (GTR) rates with minimal complications. Eden III dumbbell tumors benefited from a combined neurosurgical–thoracic approach (LCT + VATS), which resulted in higher GTR rates (100% vs. 62%, *p* < 0.01) and lower dural complications compared to neurosurgical resection alone. Eden IV extraforaminal schwannomas were best managed with VATS, which was associated with lower intraoperative blood loss (*p* = 0.018), shorter surgical duration (*p* = 0.027), and reduced postoperative complications compared to open thoracotomy. Our findings confirm that minimally invasive techniques, particularly VATS and combined neurosurgical–thoracic approaches, optimize tumor resection while reducing morbidity. However, feasibility depends on institutional resources and multidisciplinary collaboration. **Conclusions:** This study provides a stratified comparison of surgical approaches tailored to Eden classification, aiming to identify the most effective and least morbid strategies for each lesion type. Future prospective studies should validate these findings, integrating preoperative functional assessments and long-term follow-up to better stratify surgical risk, personalize operative planning, and refine surgical decision making for thoracic schwannomas.

## Simple Summary

Thoracic schwannomas are benign tumors arising from spinal nerve sheaths with potential neurological and respiratory implications depending on their location. The optimal surgical strategy remains debated, particularly regarding the balance between the extent of resection; complication rates; and postoperative morbidity. This retrospective multicenter study analyzed 106 cases classified according to the Eden system to evaluate surgical outcomes and complications associated with different techniques. Our findings suggest that minimally invasive approaches, such as video-assisted thoracoscopic surgery for extraforaminal lesions and combined neurosurgical–thoracic approaches for dumbbell tumors, improve resection rates while reducing surgical morbidity. Conversely, isolated neurosurgical approaches and open thoracotomy may be associated with increased complication risks. These results support a tailored, multidisciplinary approach based on tumor extension and anatomical constraints to optimize patient outcomes.

## 1. Introduction

Thoracic schwannomas are benign, slow-growing nerve sheath tumors that account for approximately 25% of all spinal schwannomas and 10% of posterior mediastinal masses [1]. They are primarily located in the thoracic spinal canal and the posterior mediastinum, and originate from the Schwann cells of the spinal nerve roots [2]. These tumors are classified according to the Eden system [3,4] based on their anatomical location and extension, with dumbbell schwannomas representing 15–20% of the cases due to their foraminal involvement and extension into both the spinal canal and thoracic cavity [2].

The surgical management of thoracic schwannomas remains debated, with various approaches described [5]. Bilateral posterior laminectomy and hemilaminectomy are standard for intradural and extradural lesions confined to the spinal canal or with minor lateral extension within the conjugation foramina, while minimally invasive techniques, including video-assisted thoracoscopic surgery (VATS) and combined neurosurgical–thoracic approaches, have been increasingly adopted among spine and thoracic surgeons for the treatment of dumbbell and large paravertebral lesions [6,7]. Single-stage posterior and thoracic combined procedures have been recently proposed to optimize gross total resection (GTR) rates while minimizing morbidity [8,9,10].

Another important consideration in thoracic schwannoma management is the role of long-term follow-up and recurrence rates. Although GTR is the goal, residual tumor remnants in cases of subtotal resection can lead to delayed recurrence and the need for reoperation [8]. Factors such as tumor location, surgeon experience, and patient comorbidities may influence the extent of resection achieved [9]. Understanding these predictors can further aid in tailoring surgical strategies and postoperative surveillance protocols to ensure optimal patient outcomes [8].

Previous studies have analyzed different surgical strategies for thoracic schwannomas, but a general consensus on the most effective approach has yet to be reached [5,11]. Furthermore, while surgical techniques have been extensively discussed, a comprehensive evaluation of postoperative complications remains lacking. Furthermore, several studies analyzed comprehensively all spinal schwannomas, not focusing only on the thoracic segment, which remains a complex anatomical area [9]. Among the others, the narrower canal, the different vascularization, the complexity of the mediastinal anatomy, the relatively lower experience in neurosurgery compared to the lumbar and cervical spine, and the different spinal instability contribute to the complexity of the neurosurgical management in this area [12]. Therefore, substantial and solid evidence in the literature lacks focus on these lesions.

This study is a retrospective, multicenter, observational analysis designed to assess both surgical outcomes and associated complications of different approaches tailored for different thoracic schwannomas classes. By comparing two surgical approaches for each Eden class, this research proposes to delineate the optimal strategies to maximize resection while minimizing morbidity, trying to delineate neurosurgical guidelines for thoracic schwannomas surgical management.

## 2. Materials and Methods

### 2.1. Study Design and Patient Selection

This retrospective, multicenter observational study included enrolled all patients who had undergone surgical resection of thoracic schwannomas between January 2011 and September 2024. The patients were classified according to the Eden system into four types: Type I (intradural–extradural), Type II (foraminal–paraforaminal), Type III (dumbbell-shaped), and Type IV (extraforaminal). Data were collected from electronic institutional databases across four neurosurgical centers. All the surgical procedures included in this study were performed in the neurosurgical and thoracic surgery units of the four participating institutions between 2011 and 2024. The study adhered to the Strengthening the Reporting of Observational Studies in Epidemiology (STROBE) guidelines [13].

### 2.2. Inclusion and Exclusion Criteria

Adult patients (≥18 years) with histologically confirmed thoracic schwannomas, either benign or malignant (melanotic), were included. Preoperative imaging (MRI or CT) and clinical assessments were required for inclusion, along with at least six months of postoperative clinical and radiological follow-up. The exclusion criteria encompassed patients with a diagnosis of neurofibroma, schwannomatosis, or neurofibromatosis types I and II, as well as those with incomplete or unreliable clinical records or insufficient follow-up. Clinical, surgical, and radiological records were retrieved; classified into Eden types; listed in Excel files; and then statistically inquired for significant findings to guide the surgeon to the optimal management strategy.

### 2.3. Surgical Approaches

The surgical strategy was tailored to the Eden type and location. For this purpose, for each class, two surgical approaches were compared:

- Type I: Laminectomy (LCT) or hemilaminectomy (HLCT).
- Type II: LCT or transpedicular (TPD) approach.
- Type III: LCT/TPD or a combined approach with LCT + VATS.
- Type IV: VATS or open thoracotomy (OT).

The selection of surgical approach for each patient was determined by the operating surgeon based on a combination of radiological findings, anatomical considerations, and institutional resources. The key factors influencing the decision included the Eden classification, the extent of intraspinal and extraforaminal tumor involvement, proximity to vascular or thoracic structures, and patient-specific comorbidities. Moreover, complex surgical decisions were made based on tumor extension, anatomical considerations, and surgeon expertise. Multidisciplinary discussion with thoracic surgeons was undertaken in all the cases where VATS or thoracotomy was considered. Ultimately, the choice of approach reflected a balance between maximal safe resection, minimization of perioperative morbidity, and the expertise available at each center. Intraoperative monitoring with somatosensory- and motor-evoked potentials was routinely employed and subsequently inquired for complications.

### 2.4. Outcome Measures and Follow-Up

Primary outcomes included the extent of resection and the postoperative surgical complications.

Postoperative complications were classified using the Clavien–Dindo system, which grades severity based on required interventions [14]: Grade I–II (minor, requiring pharmacological treatment or monitoring), Grade III (requiring surgical, endoscopic, or radiological intervention), Grade IV (life-threatening, requiring intensive care), and Grade V (patient death).

Secondary outcomes comprised intraoperative blood loss (g/dL Hb), operative time (minutes), postoperative hospital stay (days), and recurrence rates. Recurrences were evaluated radiologically during follow-up visits at 6 months and subsequently every 6 to 12 months based on clinical indication and imaging findings. Intraoperative blood loss was estimated based on perioperative hemoglobin concentration drop (g/dL), rather than mL volume, to better reflect the clinical impact of bleeding and minimize bias from non-standardized collection techniques.

All the patients underwent structured follow-ups at 1 month, and subsequently at 6 months postoperatively, with assessments of the neurological and respiratory functions, radiological imaging studies, and pain/motor function evaluations.

### 2.5. Statistical Analysis

Descriptive statistics were applied to summarize demographic and clinical characteristics between and within the different Eden cohorts, respectively, through the Kruskal–Wallis test and the Fisher “Exact” test for categorical and Mann–Whitney U for continuous variables. The Shapiro–Wilk was used to assess the normality of data distribution. The Mann–Whitney U was used for non-normal comparisons within the Eden classes.

A *p*-value < 0.05 was considered statistically significant.

## 3. Results

### 3.1. Sample’s Distribution

One hundred and six patients (106 pts) were included in the study, as they matched the inclusion criteria, classified according to Eden classification as Type I (*n* = 28), type II (*n* = 26), type III (*n* = 24), and type IV (*n* = 28). The sample included 50 men and 56 women; the mean age at surgery was 49.0 years (±13.9), with no significant difference between the four cohorts (*p* = 0.12). Sex distribution was similarly comparable (*p* = 0.20) (Table 1).

#### 3.1.1. Tumor Localization and Clinical Presentation

Most thoracic lesions were located at the lower (9–T12, 44.3%) and middle thoracic segment (T5–T8, 34%), with a smaller percentage involving the upper thoracic spine (T1–T4, 20.7%). The most frequent onset symptoms were low back pain (29.2%), myelopathy (27.4%), and dyspnea (12.5%), while 29 cases (27.4%) were incidentally diagnosed. Understandably, the symptoms were significantly associated with Eden type (*p* < 0.01), with dyspnea exclusively reported in Type IV tumors (13 cases, 46.4%), and myelopathy most frequently observed in Type I and III tumors.

#### 3.1.2. Surgical and Postoperative Outcomes

The overall mean intraoperative blood loss was 1.01 g/dL, showing a significant difference across Eden types, with the highest values observed in Type II tumors and the lowest in Type IV.

The mean surgical duration was 176.5 ± 67.1 min, with Type III tumors requiring the longest operative time and Type IV the shortest.

The mean postoperative length of stay was 5.27 ± 1.74 days, with no significant differences among the groups.

Overall, 35 postoperative complications were recorded in the entire cohort (33.0%), categorized according to the Clavien–Dindo classification as follows: 20 Grade I (19.0%), 8 Grade II (7.5%), 4 Grade III (3.8%), 2 Grade IV (1.9%), and 1 Grade V (0.9%).

The distribution of complications by Eden type showed the highest rate in Eden III tumors (45.8%, *n* = 11), followed by Eden II (30.8%, *n* = 8), Eden IV (25.0%, *n* = 7), and Eden I (14.3%, *n* = 4).

The most common Grade I complications included prolonged postoperative pain, transient radiculopathy, and mild pleural effusions. Grade II complications involved urinary retention and wound dehiscence requiring medical treatment. Grade III events included cerebrospinal fluid leak and pseudomeningocele requiring drainage or lumbar puncture, while Grade IV complications consisted of respiratory failure or pneumonia requiring intensive care management. The single Grade V complication occurred in a patient with an Eden IV lesion, who died intraoperatively due to acute cardiac failure following tumor decompression.

#### 3.1.3. Extent of Resection and Recurrence

GTR was achieved in 97 patients (94%), with significant variability across Eden types (KW = 10.87, *p* = 0.01). Type I and IV tumors had a 100% GTR rate, while Type III tumors had the lowest rate (75%), reflecting their anatomical complexity. Tumor recurrence occurred in 20 cases (18.9%), without a significant association with Eden classification (KW = 6.47, *p* = 0.09).

A total of 21 procedures (19.8%) were performed using a multidisciplinary surgical approach involving both neurosurgical and thoracic surgical teams. These included 11 cases of Eden type III (combined LCT + VATS) and 10 cases of Eden type IV (VATS or OT), reflecting the anatomical complexity and mediastinal involvement typical of these lesions.

Across the entire cohort, nine surgeons—including both neurosurgeons and thoracic surgeons—served as primary operators. All the other co-authors contributed to the procedures as second surgeons, ensuring a consistent technical approach and supporting the reproducibility and inter-institutional homogeneity of the surgical strategies adopted.

### 3.2. Eden Type I Results

A total of 28 patients were classified as Eden Type I, with a mean age at surgery of 53.88 ± 12.53 years. Most lesions were located at the lower thoracic level (T9–T12, 60.7%), with a predominance on the left side (67.9%). The most common presenting symptom was low back pain (39.3%), followed by myelopathy (39.3%), while 21.4% of the cases were incidentally diagnosed (Table 2).

#### 3.2.1. Surgical Approach and Operative Outcomes

Surgical management included 12 HLCT and 16 LCT. The HLCT patients had significantly higher intraoperative blood loss compared to LCT, while surgical duration was comparable between the two approaches. The postoperative length of stay was slightly longer in LCT compared to HLCT, but this difference was not statistically significant.

GTR was achieved in all the cases (100%), with tumor recurrence observed in three patients (10.7%).

#### 3.2.2. Complications

Postoperative complications were observed in four patients (14.3%), with a higher incidence in LCT patients. Grade I complications included prolonged postoperative pain (one HLCT) and transient radiculopathy (two LCTs). One LCT patient developed urinary retention requiring catheterization (Grade II). No Grade III-V complications were reported.

### 3.3. Eden Type II Results

A total of 26 patients were classified as Eden Type II, with a mean age at surgery of 43.66 ± 16.65 years. Lesions were predominantly located at the lower thoracic spine (T9–T12, 50%), with 57% occurring on the right side. The most common presenting symptom was low back pain (57.7%), followed by myelopathy (23.1%), while 19.2% of the cases were incidentally diagnosed (Table 3).

#### 3.3.1. Surgical Approach and Operative Outcomes

Surgical management included 17 LCT and 9 TPD. Blood loss was comparable between LCT and TPD, while TPD was associated with significantly longer surgical duration. The postoperative length of stay did not differ significantly between approaches.

GTR was achieved in 88% of the cases, with a higher rate in TPD (100%) compared to LCT (82%). Tumor recurrence occurred in six patients (23%), with four cases following LCT and two cases following TPD.

#### 3.3.2. Complications

Postoperative complications were recorded in eight patients (30.8%). Grade I complications included mild transient radiculopathy (three LCTs and two TPDs), while Grade II complications (one LCT and one TPD) involved urinary retention requiring catheterization. One TPD patient developed a Grade III complication (postoperative pseudo meningocele requiring drainage). No Grade IV or V complications were observed.

### 3.4. Eden Type III Results

A total of 24 patients were classified as Eden Type III, with a mean age at surgery of 47.92 ± 16.35 years. Lesions were most located at the lower thoracic spine (T9–T12, 45.8%), with a balanced left-right distribution (50%). The most frequent presenting symptom was myelopathy (37.5%), followed by low back pain (20.8%), while 29.2% of the cases were incidentally diagnosed (Table 4).

#### 3.4.1. Surgical Approach and Operative Outcomes

Surgical management included 13 exclusive neurosurgical procedures and 11 LCT combined with VATS.

Blood loss was comparable between the two groups. However, surgical duration was significantly longer for LCT/TPD. The postoperative length of stay did not differ significantly.

GTR was achieved in 75% of the cases, the lowest rate among all the Eden types, primarily due to the anatomical complexity of these tumors. GTR was higher in LCT + VATS (100%) compared to LCT/TPD (62%). Tumor recurrence occurred in seven cases (29.2%), with comparable rates in LCT/TPD (three cases) and LCT + VATS (four cases).

#### 3.4.2. Complications

Postoperative complications occurred in 11 patients (45.8%), with a higher incidence in LCT/TPD. Grade I complications included prolonged postoperative pain (three LCT/TPD and two LCT + VATS) and transient radiculopathy (two LCT/TPD and one LCT + VATS). Grade II complications included wound dehiscence requiring antibiotic therapy (one LCT/TPD and one LCT + VATS). Two patients in the neurosurgery alone group developed Grade III complications (CSF leak requiring lumbar drainage, postoperative pseudo meningocele requiring drainage), while one patient in the LCT/TPD + VATS group experienced a Grade IV complication, namely a postoperative pneumonia requiring intensive respiratory therapy. No Grade V complications were observed.

### 3.5. Eden Type IV Results

A total of 28 patients were classified as Eden Type IV, with a mean age at surgery of 50.96 ± 11.89 years. Lesions were predominantly located at the middle (T5–T8, 39.3%) and lower thoracic spine (T9–T12, 25%), with 60.7% occurring on the left side. Dyspnea was the most frequent presenting symptom (46.4%), followed by low back pain (28.6%), while 14.3% of the cases were incidentally diagnosed (Table 5).

#### 3.5.1. Surgical Approach and Operative Outcomes

Surgical management included 19 VATS procedures and 9 OTs. Both blood loss and surgery duration were significantly greater in the OT group, consistent with the more invasive nature of the procedure. The postoperative length of stay was comparable between the groups.

Gross total resection (GTR) was achieved in nearly all Type IV cases (89%), with four equally distributed recurrences.

#### 3.5.2. Complications

Postoperative complications occurred in seven patients (25%), with a higher severity in Open Thoracotomy patients. Grade I complications included mild pleural effusion (two VATS and one Open Thoracotomy). Grade II complications (one VATS and one Open Thoracotomy) involved prolonged air leak requiring extended chest tube drainage. One patient in the VATS group developed a Grade III complication (postoperative pneumothorax requiring pleural drainage), while one patient in the Open Thoracotomy group experienced a Grade IV complication (postoperative respiratory failure requiring intensive care). One OT patient died intraoperatively due to acute heart failure, one minute and a half after the 17 cm tumor removal (Grade V).

## 4. Discussion

Thoracic schwannomas are the most common benign nerve sheath tumors of the spine, often presenting as slow-growing, well-encapsulated lesions with a predilection for the posterior elements and intervertebral foramina [7,15]. While their clinical course is generally indolent, tumor growth may lead to progressive neurological deficits, foraminal compression, and, in some cases, significant extraforaminal extension, necessitating surgical intervention [8]. The choice of surgical approach is influenced by tumor size, location, and extradural extension, with the goal of achieving GTR, while minimizing perioperative morbidity and neurological impairment [16].

Despite the benign nature of thoracic schwannomas, their variable anatomical presentation poses significant surgical challenges, particularly in lesions extending beyond the spinal canal [17]. While several studies have described surgical techniques for schwannoma resection, there remains no consensus on the optimal approach for each tumor class, leading to variability in clinical outcomes and complication rates [12]. By systematically analyzing the impact of tumor location on surgical strategy and postoperative results, this study aims to bridge this gap and provide evidence-based guidance for the tailored management of these lesions.

For this purpose, this multicenter retrospective research provides a comprehensive analysis of the surgical management of thoracic schwannomas, with a particular focus on the impact of Eden classification on clinical presentation, surgical strategy, and postoperative outcomes. Our findings reinforce the intuitive conception that tumor location and extradural extension play a critical role in determining the optimal approach and expected outcomes.

Across the 106-patient cohort, we observed distinct patterns of symptomatology, extent of resection, and complication rates based on the Eden classification. Eden Type I and IV tumors, typically confined to either the intradural space or the extraforaminal region, demonstrated the highest rates of GTR (100%), and the lowest recurrence rates. Conversely, Eden Type III tumors, characterized by a dumbbell-shaped configuration, extending both intraspinally and extraforaminally, had the lowest GTR rate (75%) and the highest recurrence rate (29.2%), highlighting the technical challenges associated with their resection [18].

Our results also emphasize the influence of the surgical approach on intraoperative parameters and postoperative recovery. Less invasive techniques, such as VATS and hemilaminectomy, were associated with shorter surgical duration and reduced blood loss, while open thoracotomy and more extensive bony resections, such as bilateral laminectomy and transpedicular approaches, resulted in longer operative times and increased morbidity [15,19]. However, despite these differences, the postoperative length of stay was not significantly affected by the choice of surgical approach, suggesting that the overall burden of surgery remains relatively homogeneous across Eden types [18,20,21].

Complication rates were generally low, in line with the previous literature on schwannoma resection. The highest complication incidence (45.8%) was observed in Eden Type III tumors, where extensive foraminal involvement and epidural extension may predispose to dural tears, cerebrospinal fluid (CSF) leaks, and transient neurological deficits. Notably, Eden Type IV tumors, which are often considered technically challenging due to their extraforaminal growth, had a lower complication rate (25%), particularly in cases managed via VATS rather than open thoracotomy [15,22].

The results not only pose challenges to be answered, but give some interesting features to interpret, considering the recent history of neurosurgery and thoracic surgery. In our opinion, each Eden class is a different disease to be surgically treated and deserves separate dissertations [8].

**Figure 1:**
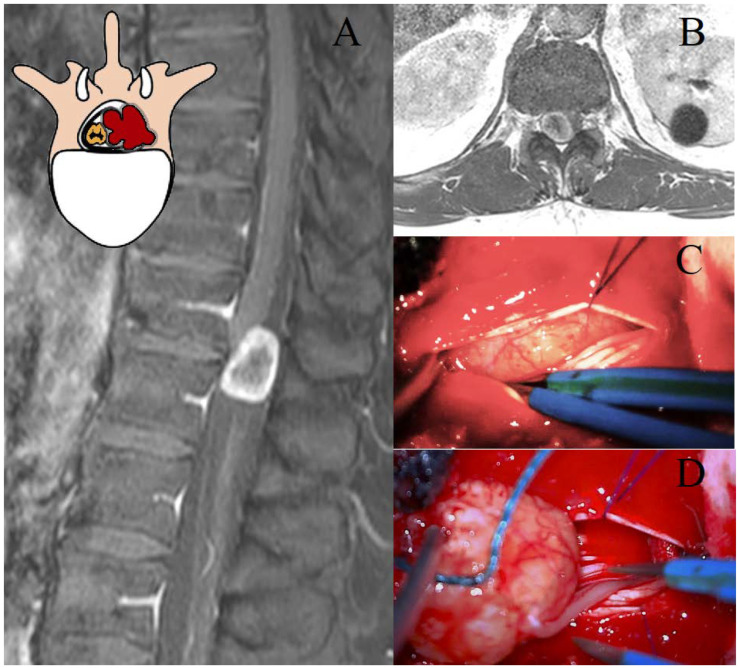
Representation of a T11–T12 thoracic schwannoma, Eden Type I. (**A**) Preoperative sagittal T1-weighted MRI with contrast, highlighting an intradural-extramedullary lesion at the T11–T12 level, located in the midline and right paramedian region, with peripheral contrast enhancement. (**B**) Axial scan showing the localization of the lesion. (**C**) Intraoperative view of dural exposure after laminectomy, with a detail of the lesion. (**D**) Microsurgical dissection and tumor removal, with identification of the thoracic nerve root of origin, which will be sacrificed.

The Eden class I tumors, the intradural–intracanal lesions, were treated by HLCT or LCT. Recently, HLCT has been increasingly performed as a neurosurgical approach for these lesions, more than LCT [23], possibly due to its perceived advantages: it is considered less invasive, requires a shorter operative time [24], and does not lead to spinal instability, despite being more uncomfortable for the surgeon [25]. Our results, despite not considering spinal instability, suggest HLCT and LCT to be comparable for invasiveness, blood loss, and surgical time. A major limitation of our study is the lack of a direct evaluation of postoperative spinal instability. Noteworthy, in our multicenter series, no patients were primarily or secondarily stabilized. It is important to note that the concept of spinal stability in the thoracic region differs from that of the lumbar and cervical spine [26]. However, in our opinion, these two techniques are substantially equivalent for the surgical management of thoracic schwannomas. The softness of the lesion allows for both ‘en bloc’ and piecemeal resection, with the main structure to be preserved being the spinal cord, which at the thoracic level permits only a gentle retraction (Figure 1) [20,27,28]. Accordingly, we advocate for the use of the traditional bilateral LCT for these lesions. Further larger and prospective studies are needed to better elucidate the impact of these surgical techniques on thoracic spinal stability.

**Figure 2:**
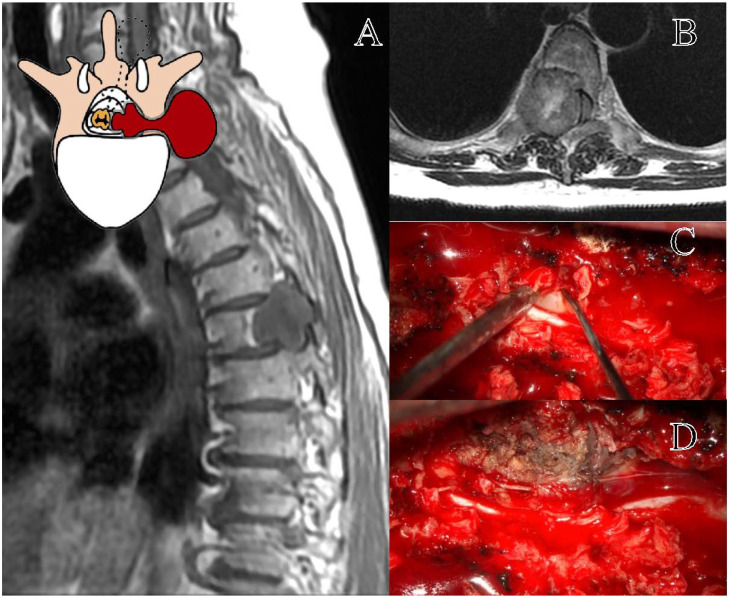
Representation of a T5–T6 schwannoma, Eden Type II. (**A**) Preoperative sagittal T1-weighted MRI with contrast, showing a lesion at the right T5–T6 level with both intradural-extramedullary and foraminal extension. (**B**) Axial MRI demonstrating the extradural and foraminal components of the lesion, with wide erosion and remodeling of the bone vertebral structure. (**C**) Intraoperative view following a laminectomy, exposing the tumor. (**D**) The transpedicular exposition of a tumor, with the demolition of the ipsilateral pedicle and articular facet.

The Eden class II lesions are rarer and less studied in the literature [3]. There is a lack of evidence regarding their optimal management [3,8]. Due to the rarity of the pathology, these tumors are often investigated together with the cervical and lumbar ones, preventing the drafting of reliable recommendations [25]. The presence of sacrificable thoracic nerve roots and the complex anatomy of the posterior mediastinum justify considering these lesions as a distinct entity [7]. In our opinion, the perception of spinal instability in these lesions warrants further investigation [26]. In our study, bilateral LCT and TPD were the investigated approaches. TPD presumably required significantly longer surgical times to create larger surgical corridors [19,29], yet did not result in substantially higher GTR rates compared to LCT (100% vs. 88%). This aspect remains an open question. Further well-structured and focused studies are needed to determine the most appropriate surgical approach for these rare Eden class II lesions. Furthermore, the resection of the ipsilateral pedicle and articular facet would presumably raise concerns regarding thoracic spinal instability (Figure 2) [19,29]. Therefore, while our study describes the enrolled cases, it does not allow us to definitively advocate the best surgical approach for these lesions. The need for secondary stabilization has been explored in studies on thoracic disk herniations [19,27]; however, these findings may not be directly applicable to intradural tumors [16].

Eden class III lesions, or dumbbell tumors, represent a distinct surgical challenge due to their dual intraspinal and paravertebral extension [21]. Their management requires a tailored approach that ensures both adequate exposure and preservation of neurological structures, particularly in the presence of foraminal and mediastinal involvement [30]. Given these complexities, a combined neurosurgical and thoracic approach has been increasingly advocated to optimize tumor resection while minimizing morbidity [2,4,18]. In our study, two strategies were compared: exclusive neurosurgical resection (LCT/TPD) and a combined neurosurgical–thoracic approach with LCT + VATS [7]. The neurosurgical-only approach was associated with longer surgical times, lower GTR rates (62% vs. 100%, *p* < 0.01), and higher rates of dural complications, likely due to limited access to the extraforaminal component [8]. Conversely, the LCT + VATS approach enabled more effective tumor removal with comparable intraoperative blood loss and a significantly higher GTR rate, suggesting a clear advantage in complex cases requiring wider exposure. Despite these benefits, the postoperative length of stay did not differ significantly between the two groups, reflecting a similar overall surgical burden.

**Figure 3:**
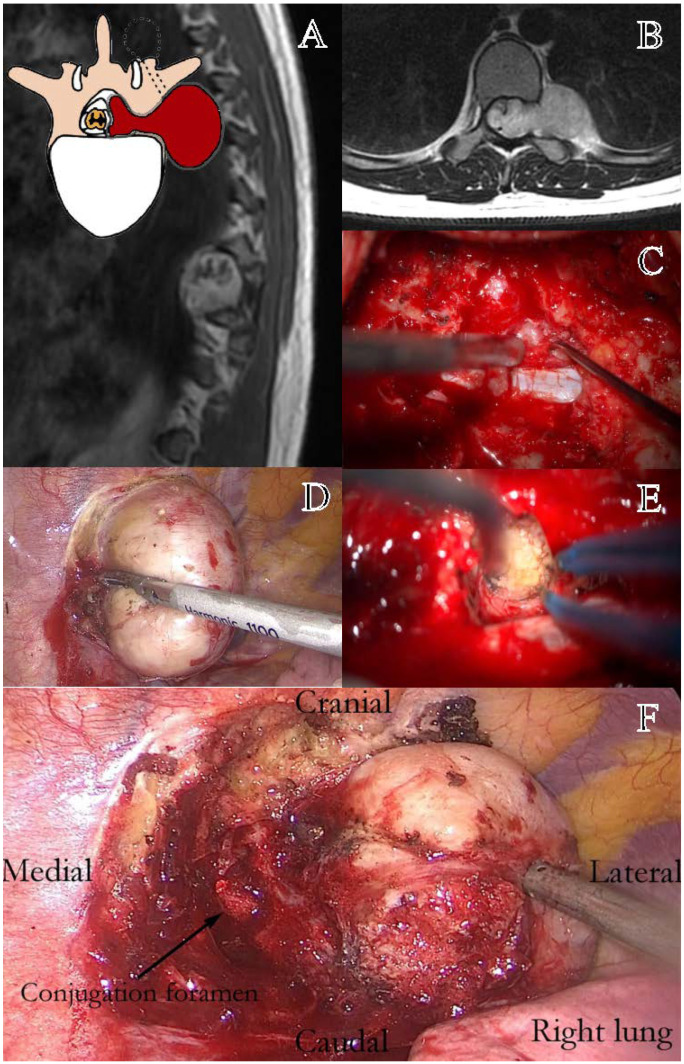
Representation of a thoracic schwannoma, Eden Type III. (**A**) Preoperative sagittal T1-weighted MRI with contrast, showing a dumbbell-shaped lesion at the T7–T9 level, located in the left paramedian and intraforaminal region, with both intradural-extramedullary and significant paravertebral extension. (**B**) Axial MRI demonstrating the intraspinal, foraminal, and large paravertebral components of the lesion. (**C**) Intraoperative view following bone removal, exposing the intradural portion of the tumor. (**D**) Thoracoscopic-assisted dissection of the paravertebral component and the vascular pedicle of the lesion. (**E**) Resection of the foraminal component, with identification of the conjugation foramen. (**F**) Final surgical field during thoracoscopic tumor resection, showing the decompressed conjugation foramen (Black arrow).

A simultaneous combined approach, as proposed in the recent literature, may further enhance surgical outcomes by ensuring superior control of both hemostasis and dural integrity [1,7]. The synchronous execution of the neurosurgical and thoracic phases allows for the direct management of epidural venous bleeding from both intraspinal and extraforaminal components, while also facilitating the timely repair of dural defects, thereby reducing the risk of cerebrospinal fluid leakage and its associated complications [7,10,15]. Although not representing a novel surgical technique per se, the synchronous single-stage combined neurosurgical and thoracoscopic approach (LCT + VATS) employed in our Eden III cohort reflects a recent and promising advancement in the management of complex dumbbell tumors. Its increasing adoption in high-volume centers, including ours, supports its inclusion as an emerging standard in appropriately selected cases. Additionally, this approach provides the optimal management of spinal stability, as the controlled and sequential resection minimizes excessive bony removal while allowing for the immediate intraoperative assessment of potential instability, reducing the need for secondary stabilization procedures (Figure 3).

However, despite its advantages, the combined approach presents limitations that must be considered. First, it requires a dedicated thoracic surgery team, making it less accessible in centers without a multidisciplinary collaboration [20]. Additionally, advanced anesthetic management is crucial, particularly for the induction of alternating pneumothorax, which facilitates thoracoscopic access while ensuring adequate ventilation [22]. Finally, patient positioning plays a critical role, as an optimal setup must allow both neurosurgical and thoracic access without compromising stability or surgical exposure. These factors highlight the need for careful patient selection and institutional resources when planning a combined LCT + VATS approach.

These findings reinforce the role of a multidisciplinary and minimally invasive strategy in the management of Eden class III tumors. While the LCT + VATS approach appears to offer clear advantages in terms of resection rates, complication reduction, and spinal stability, its feasibility depends on the availability of specialized surgical and anesthetic teams. Further prospective studies are warranted to assess its long-term impact on neurological function, recurrence rates, and overall patient recovery.

Eden class IV schwannomas, or exclusively extraforaminal tumors, represent a distinct subset of thoracic nerve sheath tumors, requiring a primarily thoracic surgical approach due to their location entirely within the posterior mediastinum [20]. Unlike other Eden types, neurosurgical involvement is generally limited to cases with foraminal encroachment [25]. The surgical strategy is dictated by tumor size, proximity to vascular and pulmonary structures, and the extent of adhesions to surrounding tissues [5]. In our study, two strategies were compared: VATS and OT [22]. The former demonstrated significantly lower intraoperative blood loss (*p* = 0.018), shorter surgical times (*p* = 0.027), and reduced postoperative complications compared to the latter, supporting its role as the preferred approach where feasible. GTR was achieved in the totality of cases managed by VATS, while OT achieved similar resection rates but with higher morbidity. However, despite these advantages, postoperative hospital stay was not significantly different. The choice between VATS and OT mainly depends on tumor characteristics [22]. While VATS is associated with lower surgical impact, it may be inadequate for large tumors, significant adhesions, or cases requiring ‘en bloc’ resection with vascular control. In such scenarios, thoracotomy provides superior exposure, facilitating safer resection and vascular management [31,32].

Despite the established benefits of VATS over OT, the impact of these techniques on postoperative pulmonary function remains poorly studied [33,34]. There are no systematic evaluations of pre- and postoperative pulmonary function tests in patients undergoing schwannoma resection. Given that both approaches involve lung manipulation, retraction, or even partial resection, perioperative respiratory function must be assessed more comprehensively [35,36]. Global spirometry, arterial blood gas analysis, and six-minute walk tests should be integrated into the preoperative evaluation and postoperative follow-up [37]. For patients with pre-existing pulmonary conditions such as COPD, asthma, or neuromuscular disorders, additional evaluations such as nocturnal cardio-respiratory monitoring may be required to assess obesity hypoventilation syndrome and post-surgical ventilatory impairment [37,38]. These assessments could help determine which surgical approach has the least impact on respiratory function and identify patients at higher risk for postoperative complications.

**Figure 4:**
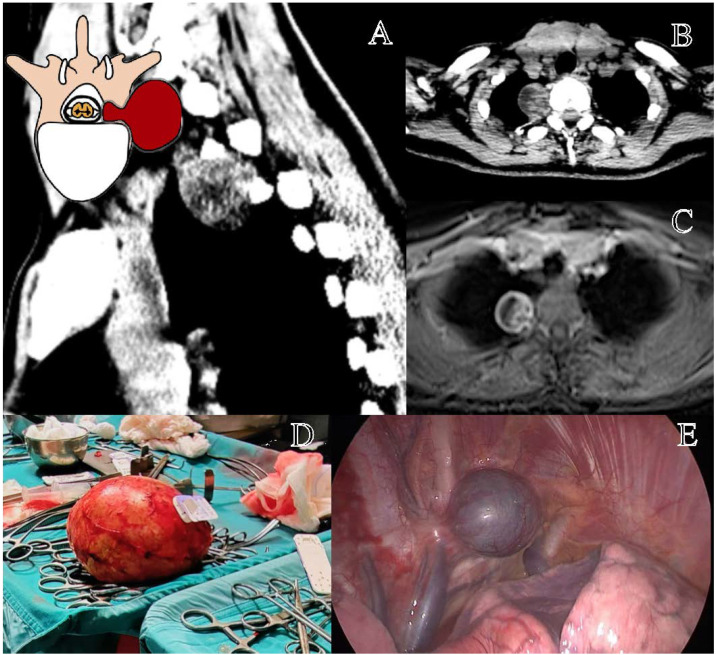
Representation of a T3–T4 right schwannoma, Eden Type IV. (**A**) Preoperative sagittal computed tomography (CT) scan showing a large paravertebral mass at the cervicothoracic junction, with foraminal extension. (**B**) Axial CT scan highlighting the tumor’s extension into the thoracic cavity. (**C**) Axial contrast-enhanced MRI demonstrating a well-circumscribed lesion with both foraminal and extensive extraforaminal growth. (**D**) Macroscopic view of the largest excised tumor specimen in our series (Dmax 17 cm). (**E**) Thoracoscopic view of the paravertebral component before resection.

The primary postoperative risks in thoracic schwannoma surgery include pneumonia and atelectasis, often due to insufficient mobilization and ineffective respiratory physiotherapy [39]. Acute respiratory distress syndrome and respiratory insufficiency are potential complications [39]. Pleural complications, including pneumothorax and chylothorax, which, if untreated, carry high morbidity, should also be considered [38,39]. Beyond pulmonary concerns, cardiovascular implications must also be considered, especially in large tumors with mediastinal compression (Figure 4) [36,38,39]. Surgical excision may lead to cardiac compression syndromes due to tumor mass effect and patient positioning, arrhythmias, and hemodynamic instability, particularly during tumor decompression, and in extreme cases, acute intraoperative cardiac arrest following sudden decompression of the heart and great vessels [17], as it happened to the unique death in our series (Figure 4D).

A multidisciplinary approach is essential for optimizing outcomes. Key elements of preoperative planning include comprehensive pulmonary function testing; embolization of highly vascularized tumors where indicated to reduce intraoperative bleeding; advanced anesthetic management, particularly for alternating pneumothorax induction to facilitate surgical access while maintaining oxygenation; and patient positioning optimization to ensure adequate exposure for both thoracic and neurosurgical teams.

Despite advancements in surgical techniques, several gaps remain in the literature [5,8,9,20,21,22,34]. There is a lack of data on postoperative pulmonary function outcomes, particularly in VATS vs. OT comparisons, limited research on the long-term respiratory impact of schwannoma resection and its effect on quality of life, and the absence of standardized preoperative functional assessment protocols, which could improve surgical decision making. These findings emphasize that VATS should be the preferred approach where feasible, due to its reduced invasiveness, shorter hospitalization, and lower incidence of postoperative pain [1,5,7,22,31,39]. However, OT remains indispensable in cases requiring complex vascular dissection, extensive adhesiolysis, or ‘en bloc’ tumor excision with lung parenchyma removal. Future studies should aim to standardize functional assessments and further explore the long-term impact of surgical approach selection on respiratory function and postoperative recovery.

Despite the valuable insights provided by this study, certain limitations must be acknowledged. As a retrospective multicenter observational study, it is subject to selection bias and variability in surgical decision making across different centers. The lack of randomization and standardized preoperative assessment protocols may influence the interpretation of outcomes, particularly regarding recurrence rates and complication profiles. Another critical limitation is the absence of systematic pre- and postoperative pulmonary function evaluations, particularly in patients undergoing VATS versus open thoracotomy for Eden class IV tumors. Future prospective studies should incorporate spirometry, arterial blood gas analysis, and structured functional assessments to better determine the impact of different surgical approaches. Additionally, while this study confirms the efficacy of minimally invasive approaches, the availability of specialized thoracic surgery teams and advanced anesthetic management remains a constraint in certain centers, limiting the generalizability of these findings. Moreover, the study does not provide long-term follow-up data on recurrence patterns or the need for secondary interventions, which should be addressed in future research.

These limitations highlight the need for prospective, multicenter trials with standardized assessment protocols to further refine surgical decision making and postoperative management for thoracic schwannomas.

## 5. Conclusions

This multicenter retrospective study analyzed 106 patients with thoracic schwannomas, classified according to the Eden system to assess surgical approaches and postoperative outcomes. Our findings suggest that surgical strategy should be tailored to tumor location to optimize resection while minimizing morbidity.

For Eden I schwannomas, LCT remains the preferred approach, ensuring adequate exposure while preserving spinal stability. Eden II tumors can be effectively managed with LCT or TPD, with no clear superiority between techniques. Eden III tumors benefit from a combined LCT + VATS approach, which achieves superior resection rates and reduces complications compared to neurosurgical resection alone. Eden IV schwannomas are best treated with VATS, which offers shorter operative time, lower blood loss, and fewer complications compared to OT.

These findings reinforce the role of a multidisciplinary and minimally invasive approach in thoracic schwannoma surgery. Future prospective studies should aim to validate these recommendations, integrating standardized preoperative assessments and long-term follow-up to further refine surgical decision making.

## Figures and Tables

**Table 1:** Demographic, clinical, surgical, and postoperative characteristics of the 106 patients included in the study stratified by Eden classification (Types I-IV).

Model	Sample (*n* = 106)	Eden Type I (*n* = 28)	Eden Type II (*n* = 26)	Eden Type III (*n* = 24)	Eden Type IV (*n* = 28)	Statistics	
Sex						KW	*p*-value
Men	50	13	12	15	10		
Women	56	15	14	9	18		
Age (yrs)	49.0 (±13.9)	53.88 (±12.53)	43.66 (±16.65)	47.92 (±16.35)	50.96 (±11.89)	5.82	0.12
Right side	53	9	15	12	17	4.63	0.20
Level							
Upper (T1–4)	22	7	6	5	4		
Middle (T5–8)	36	4	7	8	17		
Lower (T9–12)	48	17	13	11	7		
Signs and symptoms						14.72	<0.01
Incidental diagnosis	29	6	5	7	11		
Low back pain	31	11	15	5	0		
Radiculopathy	4	0	0	0	4		
Myelopathy	26	11	6	9	0		
Dyspnea	16	0	0	3	13		
Intraoperative blood loss (g/dL)	1.01 (±1.01)	1.10 (±0.67)	1.35 (±0.84)	0.81 (±0.71)	0.79 (±0.52)	9.62	0.02
Surgery Duration (mins)	176.5 (±67.1)	211.9 (±59.47)	212.7 (±80.22)	230.5 (±88.32)	104.8 (±28.7)	12.35	<0.01
Postop hospital stay (days)	5.27 (±1.74)	4.5 (±1.2)	5.5 (±1.4)	6.07 (±1.4)	5.83 (±2.14)	5.37	0.15
Surgical approach							
		HLCT 12	LCT 17	LCT/TPD 13	OT 9		
		LCT 16	TPD 9	LCT + VATS 11	VATS 19		
GTR	97 (94)	28 (100)	23 (88)	18 (75)	28 (100)	10.87	0.01
Melanotic lesions	8	2	1	3	2	3.11	0.38
Complications						8.91	0.03
Class I	20	3	5	7	5		
Class II	8	1	2	3	2		
Class III	4	0	1	2	1		
Class IV	2	0	0	1	1		
Class V	1	0	0	0	1		
Recurrences	20	3	6	7	4	6.47	0.09
Follow-up	5.98 (±2.16)	5.67 (±1.63)	6.71 (±1.95)	6.19 (±2.82)	5.26 (±2.31)	8.89	0.03

Data are presented as mean ± standard deviation for continuous variables and absolute frequency (percentage) for categorical variables. Statistical comparisons among the four groups were performed using the Kruskal–Wallis test for continuous variables and the Chi-square test for categorical variables.

**Table 2:** Surgical and postoperative outcomes of Eden Type I patients stratified by hemilaminectomy (HLCT) and bilateral laminectomy (LCT).

Eden Type I			
Model	Hemilaminectomy (12)	Bilateral laminectomy (16)	statistics
Blood loss	1.42 (±0.64)	0.86 (±0.69)	U 41.5; *p* = 0.042
Surgery duration	219.4 (±64.31)	206.1 (±55.04)	U 61.5; *p* = 0.19
Postop hospital stay	4.3 (±1.15)	4.8 (±1.25)	U 66.5; *p* = 0.31
Extent of resection	12	16	-
Recurrences	1	2	-
Complications			KW 0.75; *p* = 0.38
Grade I	1	2	
Grade II		1	
Grade III			
Grade IV			
Grade V			

**Table 3:** Surgical and postoperative outcomes of Eden Type II patients stratified by bilateral laminectomy (LCT) and transpedicular approach (TPD).

Eden Type II			
Model	Bilateral laminectomy (17)	Transpeduncular (9)	statistics
Blood loss	1.25 (±0.38)	1.1 (±0.9)	U 58.5; *p* = 0.21
Surgery duration	168.4 (±50.8)	244.6 (±72.02)	U 42.0; *p* = 0.047
Postop hospital stay	5.96 (±2.34)	6.71 (±1.95)	U 64.0; *p* = 0.26
Extent of resection	14 (88)	9 (100)	-
Recurrences	4	2	-
Complications			KW 1.89; *p* = 0.17
Grade I	3	2	
Grade II	1	1	
Grade III		1	
Grade IV			
Grade V			

**Table 4:** Surgical and postoperative outcomes of Eden Type III patients stratified by neurosurgery alone (LCT/TPD) and neurosurgery combined with VATS (LCT + VATS).

Eden Type III			
Model	Neurosurgery alone (13)	Neurosurgery + VATS (11)	statistics
Blood loss	0.96 (±0.69)	0.77 (±0.83)	U 50.5; *p* = 0.28
Surgery duration	244.2 (±89.71)	214.8 (±87.29)	U 41.0 *p* = 0.039
Postop hospital stay	5.91 (±1.35)	6.19 (±2.82)	U 57.5; *p* = 0.15
Extent of resection	7 (62)	11 (100)	*p* < 0.01
Recurrences	3	4	
Complications			KW 2.18; *p* = 0.15
Grade I	4	3	
Grade II	2	1	
Grade III	2		
Grade IV		1	
Grade V			

**Table 5:** Surgical and postoperative outcomes of Eden Type IV patients stratified by VATS and OT.

Eden Type IV			
Model	VATS (19)	Open thoracotomy (9)	statistics
Blood loss	0.57 (±0.51)	1.61 (±0.54)	U 38.5; *p* = 0.018
Surgery duration	92.5 (±24.08)	147.3 (±30.2)	U 40.0; *p* = 0.027
Postop hospital stay	5.9 (±2.05)	5.58 (±2.23)	U 54.5; *p* = 0.24
Extent of resection	17	8	-
Recurrences	2	2	-
Complications			KW 3.12; *p* = 0.074
Grade I	3	2	
Grade II	1	1	
Grade III	1		
Grade IV		1	
Grade V		1	

## Data Availability

The data presented in this study are available upon request from the corresponding author.
